# Asphyxiating Thoracic Dysplasia: clinical and molecular review of 42 families

**DOI:** 10.1186/2046-2530-1-S1-O4

**Published:** 2012-11-16

**Authors:** V Cormier-Daire, C Huber, J Baujat, R Caumes, H Kayirangwa, M Le Merrer, KH Le Quan Sang, A Munnich

**Affiliations:** 1 Département de Génétique, INSERM U781, Hôpital Necker Enfants Malades, France

## 

Asphyxiating Thoracic Dysplasia (ATD) ((MIM 208500, MIM 6112633, MIM 613091, MIM 61819, MIM614376) belongs to the short rib polydactyly group and is characterized by a long and narrow thorax, short long bones and trident acetabular roof. Polydactyly, retinal degeneration, cystic renal and liver diseases have been occasionally reported. Today, mutations in *IFT80* (MIM 611177), *DYNC2H1* (MIM 603297), *TCC21B* (MIM 612014) and *WDR19* (MIM 608151) genes have been reported in ATD. Through a national grant (PHRC, AOM 06031), we have collected 55 ATD cases including 29 fetuses issued from 42 families who benefit the combined approach of deep phenotyping and molecular screening of *IFT80* and *DYNC2H1*. The series included 26 alive cases ranging in age from 6 months to 36 years. Respiratory treatment was needed in 46%, including positive pression respiration, and invasive or non-invasive ventilation. Cystic renal and liver diseases occur in 16% of cases; whereas retinal degeneration was present in 40 % cases aged more than 2 years (6/15). The molecular screening allowed us to detect *DYNC2H1* mutations in 63% and IFT80 mutations in 6%. In 6 cases, only one heterozygote mutation in either *IFT80* or *DYNC2H1* was identified. Finally, the two genes were excluded in 31% cases. These preliminary results emphasize that *DYNC2H1* is the major gene responsible for ATD. The presence of only one mutation (27% of mutated cases) may suggest a digenic diallelic inheritance. Ongoing studies will hopefully lead to the identification of other disease genes.

